# Identification and Control of Latent Bacteria in *in vitro* Cultures of Sweetpotato [*Ipomoea batatas* (L.) Lam]

**DOI:** 10.3389/fpls.2020.00903

**Published:** 2020-07-03

**Authors:** Myriam Lorena Izarra, Ana Luz Panta, Carmen Rosa Maza, Brenda Carina Zea, Juan Cruzado, Liliam Rosario Gutarra, Cristina R. Rivera, David Ellis, Jan Frederik Kreuze

**Affiliations:** International Potato Center (CIP), Lima, Peru

**Keywords:** 16S rRNA, endogenous microbes, tissue culture, contamination, genetic resources

## Abstract

Bacterial microorganisms which are latent in *in vitro* cultures can limit the efficiency of *in vitro* methods for the conservation of genetic resources. In this study we screened 2,373 accessions from the *in vitro* sweetpotato germplasm collection of the International Potato Center in Lima, Peru for bacteria associated with plantlets in tissue culture through a combination of morphological methods and partial 16S rDNA sequencing. Bacteria were detected in 240 accessions (10% of the accessions screened) and we were able to isolate 184 different bacterial isolates from 177 different accessions. These corresponded to at least nineteen Operational Taxonomic Units (OTUs) of bacteria, belonging to the genera *Sphingomonas*, *Bacillus*, *Paenibacillus*, *Methylobacterium*, *Brevibacterium*, *Acinetobacter*, *Microbacterium*, *Streptomyces*, *Staphylococcus*, and *Janibacter*. Specific primers were developed for PCR based diagnostic tests that were able to rapidly detect these bacteria directly from tissue culture plants, without the need of microbial sub-culturing. Based on PCR screening the largest bacterial OTUs corresponded to a *Paenibacillus* sp. closely related to *Paenibacillus taichungensis* (41.67%), and *Bacillus* sp. closely related to *Bacillus cereus* (22.22%), and *Bacillus pumilus* (16.67%). Since *in vitro* plant genetic resources must be microbe-free for international distribution and use, any microbial presence is considered a contamination and therefore it is critical to clean all cultures of these latent-appearing bacteria. To accomplish this, plantlets from *in vitro* were transferred to soil, watered with Dimanin^®^ (2 ml/l) weekly and then reintroduced into *in vitro*. Of the 191 accessions processed for bacterial elimination, 100% tested bacteria-free after treatment. It is suspected that these bacteria may be endosymbionts and some may be beneficial for the plants.

## Introduction

Sweetpotato [*Ipomoea batatas* (L.) Lam] is the sixth most important crop for global food security (CIP, 2020). Although it is a perennial vine, it is usually grown as an annual crop. The International Potato Center (CIP) maintains the global *in trust* collection of sweetpotato germplasm and the cultivated collection is maintained as clones, which is expensive and carries risks of loss due of infectious diseases or adverse weather conditions if performed in the field (Benson, 2002). One of the current forms of *ex situ* conservation of clonal germplasm is by *in vitro* tissue culture. *In vitro* conservation offers advantages such as: lack of field infections, protection against adverse environmental conditions; timely access to material, as well as the feasibility of maintaining disease-free material for propagation and export. Tissue culture also allows rapid clonal propagation of a large number of plants in a short time and in small spaces (Benson, 2002).

Prevention of microorganism contamination in plant tissue culture is essential. Plants growing *in vitro* are considered to be under stress and may be predisposed to direct infection, even by non-pathogenic bacteria (Habiba et al., 2002). Although the culture medium in which the plant tissue is cultured is a good source of nutrition for microbial growth, during micropropagation endogenous microbes can remain undetected because the concentration of salts and/or sucrose are not ideal or the pH and/or temperature are not optimal for bacterial growth. When the culture conditions change, as they do during normal plant growth, they can become more favorable to microbial growth and bacteria that were previously undetectable can multiply and even damage the growing plants (Wojtania et al., 2005). Endogenous bacteria can also be difficult to isolate and may only survive or grow well inside the plant. Even if bacteria do not cause any obvious damage, their mere presence forms an impediment to international movement of germplasm due to phytosanitary restrictions and therefore in this paper they are referred to as contaminants of the *in vitro* cultures.

Ribosomal DNA (rDNA) genes are conserved throughout all living organisms and consists of conserved regions which allows the use of universal primers to amplify them from all bacteria and highly variable regions, which enable identification of different species and their relationships based on level of sequence identity and phylogenetic analysis (Clarridge, 2004). The analysis of the 16S rDNA sequences of bacteria have been demonstrated to be particularly useful for the identification of different taxa by comparison to curated data bases (Cole et al., 2011; Poretsky et al., 2014). Its implementation is quick simple and cost-effective (Cox et al., 2019), complementing the time-consuming traditional identification methods which include phenotypic and biochemical techniques to discover novel genera and species (Woo et al., 2008). Classification by 16S rDNA sequences is, however, restricted by the sequences available in curated databases (Poretsky et al., 2014) and provides no information of functional properties, as these can vary considerably within the same genus or even species (Cox et al., 2019).

The objective of the present study was to detect the presence of, and characterize and identity to genus, bacteria present in the *in vitro* sweetpotato collection. The identification of the bacteria to the level of genus was done by amplifying and sequencing bacterial 16S rDNA but also involved the develop a highly sensitive PCR based screening method that could detect bacterial contamination in *in vitro* plants. Finally, we detail a method for complete bacterial elimination from *in vitro* sweetpotato plants and the generation of bacteria-free *in vitro* sweetpotato cultures.

## Materials and Methods

### Biological Material

*Ipomoea batatas* plants (2373 accessions) from the CIP *in vitro* genebank were selected and examined for bacterial contamination. The *in vitro* cultures were maintained on conservation medium (MCB) {2 mg/l calcium pantothenic, 100 mg/l calcium nitrate, 100 mg/l L-arginine, 200 mg/l ascorbic acid, 20 mg/l putrescine HCl, 30 g/l sucrose, 3 g/l phytagel, and 4.33 g/l Murashige and Skoog (Murashige and Skoog, 1962) basal salts [-Caisson^®^], pH 5.7} at 20 ± 1°C,16 h light photoperiod, light intensity of 40 μmol/m^2^/s in CIP’s genebank in Lima, Peru (Panta et al., 2009).

### Isolation of Bacterial From *in vitro* Plants

Two months old *in vitro* plants were used for isolating bacterial contaminants. In the first instance bacterial contamination was assessed visually by examining plants and growth media under fluorescent light. Such detection is often difficult as bacteria do not grow well in tissue culture medium and therefore may only be marginally visible as a tiny faint halo around the base or roots of the *in vitro* plant. Regardless of the ability to visually detect bacterial growth, microbial cultures were initiated from all plants by placing 1–2 mm of stem segments (cut at the base of the plant) and 5 mm of root tissue in tubes (16 mm × 150 mm) with 3 ml of modified Nutrient Broth (Atlas, 2004) (NB containing: 5 g/l peptone, 1 g/l beef extract, 5 g/l sodium chloride, 10 g/l glucose, pH 7). Each tube was incubated at room temperature (RT) for 3 days; if bacterial growth was not apparent, the tubes were incubated at 28°C for 3 additional days followed by 3 weeks at RT. The cultures that did not show obvious bacterial growth after these incubations were spread (50 μl) on: (i) modified Nutritive Agar (NA: 5 g/l peptone, 3 g/l beef extract, 2.5 g/l glucose, and 15 g/l agar, pH 7) (Atlas, 2004) and incubated at RT for 20 days, and (ii) on Kelman agar (French et al., 1995) (KA: 10 g/l peptone, 2.5 g/l C_6_H_12_O_6_, 1 g/l casamino acids [Difco^–®^] and 15 g/l agar, pH 7) and incubated at 28°C for 2 days followed by RT for 18 days.

### Morphological and Biochemical Characterization

Bacteria isolated from plant tissue cultures were subsequently grown on NA, incubated at 28°C for 2 days, and in the case of slow growing bacteria left at RT for 7 additional days. Bacteria growing on the NA plates were grouped based on the following morphological characteristics of the bacterial colonies: margin, surface structure, form, elevation, texture, color and diameter (mm). Also, the Gram reaction by Hucker’s modified method (Smibert and Krieg, 1994) was performed in addition to the following biochemical assays: oxidase (Naz et al., 2012), catalase (Macfaddin, 2000), and Oxide/fermentation (O/F) (Hijnen et al., 1995).

### 16S rDNA Sequencing and Identification

Individual colonies, from the various morphotypes were cultured in 1 ml of NB in Eppendorf tubes and incubated at 28°C for 2 days with constant agitation (124 rpm) in an incubator shaker. Slow growing bacterial cultures were left for 7 additional days at RT. One μl of each bacterial suspension was used to amplify 16S rDNA encoding sequences using primer pairs pA-pF (Edwards et al., 1989). The reaction mix contained: 125 pmol of each primer, 200 μmol of each deoxyribonucleoside triphosphate, 1X GoTaq reaction buffer (Promega^®^) and 0.625 unit of Taq DNA polymerase (Promega^®^). Sterile distilled water was added to a total volume of 25 μl. PCR amplification was carried out with a thermal cycler Verity (Applied Biosystems^®^) using the following program: initial denaturation for 3 min at 95°C, followed by 35 cycles consisting of denaturation for 30 s at 95°C, annealing for 30 s at 55°C, and DNA synthesis for 80 s at 72°C. A final extension of 3 min at 72°C was added at the end of the 35 cycles. PCR amplified fragments of the expected size were purified using the Wizard PCR purification kit (Promega^®^), and sent for direct Sanger sequencing to Macrogen (Seoul, South Korea). Similarities of the determined partial 16S rDNA gene sequences to known sequences were determined using BLAST against NCBI Genbank database^1^ as well as the Ribosomal database project (RDP)^2^. Each unique sequence was defined as representing an Operational Taxonomic Unit (OTU).

### Design of Specific Primers and Test of Specificity

16S rDNA sequences were aligned using the ClustalW algorithm as implemented in Mega 5.0 (Tamura et al., 2011) based on which regions were selected for the designing of specific forward and reverse primers for each of the identified bacterial OTUs. The melting temperatures were calculated with the software Vector NTI Advance 9 (Invitrogen^®^).

The specificity of the designed primers was evaluated by performing PCR reactions, as described previously, but with varying annealing temperatures according to the primer pair used, with all bacteria evaluated in this study as well as pure plant DNA as a negative control and extracted according to Doyle (1987).

### Sensitivity of Detection

The PCR detection threshold for the bacteria was determined using serial dilutions of each bacterium for their selected specific primers. The concentration of cells was estimated using spectrophotometry to establish the relationship between OD_600_ = 0.1 and the number of colony-forming units (cfu) per milliliter. Bacterial suspensions from 2-day-old cultures were adjusted to 10^9^ cfu ml^–1^ and tenfold serial dilutions were prepared from this. The detection was performed using two types of template: (a) a 1 μl bacterial suspension of each dilution was used as the template by direct addition to the PCR reaction, and (b) a 10-μl aliquot of each dilution was added into 1.5 ml microcentrifuge tube with 0.1 g of stem segments and root tissue of a non-contaminated *in vitro* plant which was subsequently pulverized in liquid nitrogen. DNA was then extracted (Doyle, 1987) and resuspended in 80 μl of nuclease-free water. 1 μl of DNA extract from each dilution was used as the template for PCR with the selected specific primers. Each experiment was repeated three times.

### Consistency Between Molecular and Morphological Grouping

The efficiency of the designed primers was evaluated for each group identified by conventional microbiological methods. When isolates did not amplify with the expected primer pair according to their conventionally determined groupings, these isolates were then tested with other primer pairs corresponding to isolates with similar morphological characteristics. PCR amplified products were then cloned into *Escherichia coli* using a TA Cloning Kit with pCR2.1 Vector (Invitrogen^®^) according the manufacturer’s instructions, purified and sent for sequencing to Macrogen. Additionally, the OTUs as determined by PCR were confirmed by cloning and sequencing a random sample from each OTU amplified by their specific primers. Similarities of the determined partial 16S rDNA genes to known sequences were determined using BLAST as described before. A phylogenetic tree based on the 16S rDNA gene sequences between isolates was constructed by the Neighbor Joining method in Mega 6.06 software. The consistency in groupings based on PCR (OTUs) and morphologic methods was evaluated by comparing two proportions as implemented in the function *prop*.*test* (Newcombe, 1998) of the statistical software package R (R Development Core Team, 2010).

### Bacteria Elimination and Testing

**FIGURE 1:**
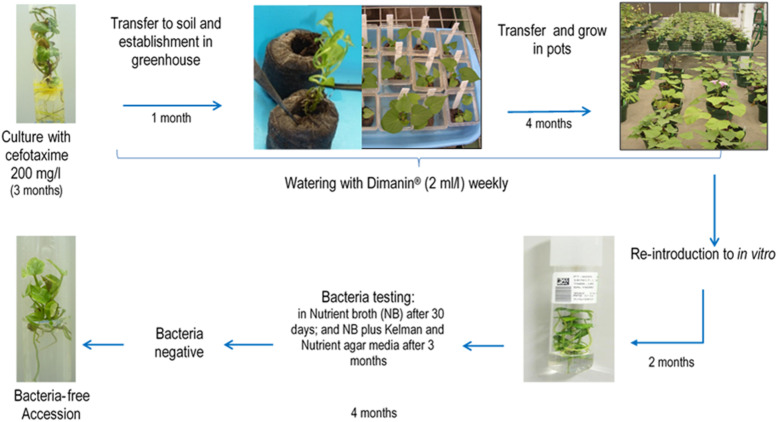
General scheme of the methodology applied at CIP for bacteria elimination in *in vitro* sweetpotato germplasm. The method consists of antimicrobial treatments *in vitro*, plant growth and antimicrobial treatment in the greenhouse and re-introduction into *in vitro*.

*In vitro* shoot cultures were grown in sweetpotato propagation medium (MPB) [Murashige and Skoog salts (Murashige and Skoog, 1962), 2.0 mg/l calcium pantothenate, 100 mg/l calcium nitrate, 100 mg/l L-arginine, 200 mg/l ascorbic acid, 20 mg/l putrescine HCl, 10 mg/l gibberellic acid, 30 g/l sucrose, and 3 g/l phytagel] supplemented with cefotaxime 200 mg/l, and grown at 24°C ± 1°C, 16 h light and 45 μmol.m^2^.sec. Plantlets with visible bacterial contamination (visible often as a very faint halo under the cut stem) showing good rooting were transferred to jiffy 7 pots^®^ and grown in a greenhouse for 1 month at 23–26°C; prior to transfer to pots containing a soil mix [moss 8%, sand 12%, humus of earthworm 72% and peat (Promix^®^) supplemented with 20-20-20 NPK, benomyl 10 g and pentachlorobenzene 10 g] and grown in the greenhouse for 4 months. Plants are watered with Dimanin^®^ (2 ml/l) weekly (Figure 1).

After the 4th month of disinfectant Dimanin^®^ treatment, plants were reintroduced into *in vitro* by taking an apical cutting (∼15 cm), excising this into single-nodal stem segments and placing these nodal segments into a container with an anti-mite and fungicide solution [Kenyo^®^ (fenpiroximato) 1 ml/l, Oberon 240 SC^®^ (spiromesifen) 20 μl/l, Farmathe^®^ (benomyl) 1g/l, and Tween 20^®^ 1 ml/l] for 15 min, followed by 5–6 rinses with tap water with vigorous agitation, a rinse with 96% ethanol for 30 s and finally rinsing with distillated water 5–6 times shaking vigorously. The nodal segments were then transferred into a sterile container containing 2.5% NaClO for 10–15 min, shaking every 5 min followed by five rinses with a sterile ascorbic acid solution (200 mg/l) with vigorous shaking. Explants were removed to a sterilize Petri dish where dead tissue was removed and the segments were placed on MPB medium, one explant per test tube and incubated in conditions indicated above. Cultures were evaluated weekly to ensure there was no visible contamination.

After 6 weeks, the explants were subcultured on MPB and checked for the presence of bacteria by taking pieces of the base, leaves and roots with a little surrounding culture medium, and placing these in a test tube (16 mm × 125 mm) containing 2 ml NB. The NB tubes were incubated at 23–25°C for 3 days, then at 28°C for other 3 days and tubes were evaluated for the presence or absence of turbidity at 3 and 6 days. Negative samples (no turbidity) were incubated for an additional 6 weeks at 23–25°C when they were again evaluated. If still negative for visible bacterial growth, the tubes were incubated for an additional 3 months, after which a second bacteria testing in NB was done in addition to streaking on Kelman Nutrient agar media. Accessions that showed no signs of bacterial contamination and continued to grow for at least another 2 months were transferred back to the basic *in vitro* collection.

The primers designed in this study were subsequently used to confirm the absence of bacteria in a subset of accessions after cleanup: *Bacillus* spp.1 (*n* = 26), *Bacillus* spp.3 (*n* = 21), *Bacillus* sp.2 (*n* = 1), *Bacillus* spp.4 (*n* = 2), *Brevibacterium* sp. (*n* = 1), *Janibacter* spp. (*n* = 5), *Methylobacterium* sp. (*n* = 1), *Paenibacillus* spp.1 (*n* = 4), *Paenibacillus* spp.2 (*n* = 55), *Sphingomonas* sp. (*n* = 1), *Staphylococcus* spp.1 (*n* = 6), *Streptomyces* sp. (*n* = 1), and *Acinetobacter* sp. (*n* = 1) (Supplementary Table S2).

## Results

### Bacterial Detection

**FIGURE 2:**
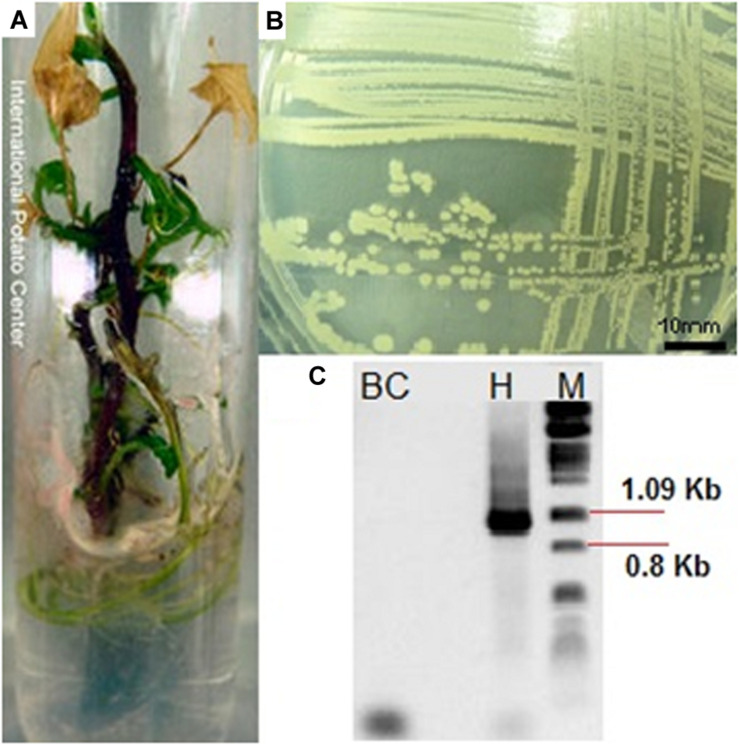
**(A)** *In vitro* sweetpotato accession (CIP 420613) showing signs of bacterial contamination, **(B)** an example of morphology of isolated bacteria, and **(C)** PCR amplification of 16S rDNA gene segments with primers pA and pF (1,066 bp) [BC, background Control, H: PCR amplification of the bacteria, M: molecular weight marker (lambda phage DNA digested with *Pst*I].

Out of the 2,373 accessions processed, 240 (10.1%) were contaminated with bacteria (Figure 2A). A total of 184 different bacterial isolates were obtained from 177 accessions because cultures from 63 accessions could not be re-isolated. 21.2% of the isolates were detected on solid (NA and KA), and 78.8% isolates were detected on liquid (NB) microbiological medium. Some of these accessions were contaminated with two different types (No. CIP 441159, 400256, 421135, 422556, and 440699), and other (N0. CIP 440473) with three types of bacteria.

### Morphological, Biochemical, and Molecular Characterization

Seventeen different bacterial groups (A–Q) were identified based on morphological and biochemical characteristics (Table 1 and Figure 2B). The morphological group “L” was the most difficult to detect because members of this group grew slowly (at least 7 days at RT to visually detectable amount), while the other groups grew rapidly (visually detectable after 2 days at 28°C). Supplementary Table S1 provides the sweetpotato accession numbers from which bacteria of the different morphological groups were identified.

**TABLE 1:** Distinctive phenotypic characteristics of the 184 strains isolated from *in vitro* cultures of *Ipomoea batatas.*

**Morphological group**	**Margin**	**Shine^a^**	**Form**	**Elevation**	**Texture**	**Color**	**Diameter (mm)**	**Gram reaction^b^**	**Cell morphology**	**Catalase**	**Oxidase**	**O/F**	**No. morphologies detected**
A	Entire	+	Circular	Convex	Viscous	Dark yellow	0.5	−	Rods	+	−	+/+	2
B	Entire	+	Circular	Convex	Viscous	Cream	1.8	+	Staphylococci	+	−	+/+	8
C	Entire	−	Circular	Flat	Viscous	Cream−white	8	+	Rods	+	+	+/+	29
D	Undulate	+	Irregular	Flat	Viscous	White−gray	2.5	±	Rods	+	+	+/+	85
E	Entire	−	Circular	Flat	Viscous	Pink	0.2	−	Coccobacilli	+	+	−/−	1
F	Undulate	+	Irregular	Flat	Viscous	Dark yellow	3	+	Rods	+	+	+/+	1
G	Entire	+	Circular	Convex	Viscous	Yellow-cream	1.1	+	Rods	+	−	±	1
H	Entire	+	Circular	Convex	Viscous	Yellow-cream	2.5	+	Rods	+	+	+/+	12
I	Entire	+	Circular	Flat	Viscous	Mustard	2	+	Rods	+	+	+/+	4
J	Entire	+	Circular	Convex	Viscous	Pale yellow	0.5	+	Rods	+	+	+/+	23
K	Entire	+	Circular	Convex	Viscous	White	2	+	Rods	+	±	+/+	1
L	Entire	−	Circular	Convex	Dry	Yellow	1.5	+	Rods	+	+	+/+	7
M	Undulate	+	Irregular	Flat	Viscous	Leaden-white	0.8	+	Rods	+	+	+/+	2
N	Entire	+	Circular	Convex	Viscous	Pale yellow	1.2	+	Rods	+	+	±	1
O	Entire	+	Circular	Convex	Viscous	Pale yellow	2.3	±	Coccobacilli	+	−	±	2
P	Entire	+	Circular	Convex	Viscous	Yellowish white	0.3	+	Cocci	+	−	−/−	4
Q	Entire	−	Circular	Convex	Dry	Pale yellow	2	+	Filamentous	+	+	+/+	1

*^*a*^Presence of shine (+) and Absence of shine (−). ^*b*^Positive reaction (+), Negative reaction (−), variable reaction (±).*

Representatives from all 17 morphological groups were used for PCR amplification of the 16S rDNA gene with primer pairs pA and pF (Figure 2C). Based on partial 16S sequence similarities 19 sequence groups (defined as OTUs) could be identified, where isolates from morphological groups “H,” “I,” and “J” resulted in very similar 16S sequences (the nucleotide difference between H and I, and between I and J was only to 0.6%). Two different OTUs were detected in each of the morphological groups “A” and “B” (see following section). Table 2 provides the Genbank accession numbers of each sequenced fragment, the result of comparison to Genbank and RDP databases and their designated OTU.

**TABLE 2:** Identification of isolated bacteria based on sequence similarity of partial 16S sequences to the NCBI and RDP databases.

**No. CIP**	**Morphological group**	**Closest relative according to Blast in NCBI (% identity)**	**Closest relative according to SeqMatch in RDP (% identity)**	**Designated OTU**	**GenBank accession no.**
420278	A	*Sphingomonas paucimobilis* (98)	*Sphingomonas paucimobilis* (100)	*Sphingomonas* sp.	KF192619
420933		*Pseudomonas oryzihabitans* (100)	*Pseudomonas oryzihabitans* (100)	*Pseudomonas* sp.	KF192618
400311	B	*Staphylococcus pasteuri* (99)	*Staphylococcus pasteuri* (99.4)	*Staphylococcus* sp.1	KF192620
422567		*Staphylococcus epidermidis* (93)	*Staphylococcus* (81.0)	*Staphylococcus* sp.2	KF192621
421426	C	*Bacillus cereus* (99)	*Bacillus cereus* (97.8)	*Bacillus* sp.1	KF192608
420251	D	*Paenibacillus taichungensis* (99)	*Paenibacillus taichungensis* (98)	*Paenibacillus* sp.1	KF192617
400108	E	*Methylobacterium extorquens* (99)	*Methylobacterium extorquens* (99)	*Methylobacterium* sp.	KF192614
442368	F	*Bacillus firmus* (99)	*Bacillus firmus* (99.5)	*Bacillus* sp.2	KF192609
442536	G	*Brevibacterium casei* (99)	*Brevibacterium casei* (98.6)	*Brevibacterium* sp.	KF192612
420613	H	*Bacillus pumilus* (99)	*Bacillus pumilus* (98.7)	*Bacillus* sp.3a	KF192611
442507	I	*Bacillus pumilus* (99)	*Bacillus pumilus* (96.6)	*Bacillus* sp.3b	KT624615
402715	J	*Bacillus pumilus* (100)	*Bacillus pumilus* (100)	*Bacillus* sp.3c	KT624616
441180	K	*Bacillus licheniformis* (99)	*Bacillus licheniformis* (98.6)	*Bacillus* sp.4	KF192610
400062	L	*Janibacter hoylei* (99)	Uncultured bacterium (100)	*Janibacter* sp.1	KF192613
400280	M	*Paenibacillus illinoisensis* (99)	*Paenibacillus illinoisensis* (95.3)	*Paenibacillus* sp.2	KF192616
440199	N	*Microbacterium paraoxydans* (98)	*Microbacterium* sp. (88.3)	*Microbacterium* sp.	KF192615
421115	O	*Acinetobacter lwoffii* (99)	*Acinetobacter lwoffii* (95.4)	*Acinetobacter* sp.	KF192607
187002.1	P	Uncultured bacterium (99)	Uncultured bacterium (99)	*Janibacter* sp. 2	KF192623
420405	Q	*Streptomyces parvulus* (99)	*Streptomyces parvulus* (95.4)	*Streptomyces* sp.	KF192622

**FIGURE 3:**
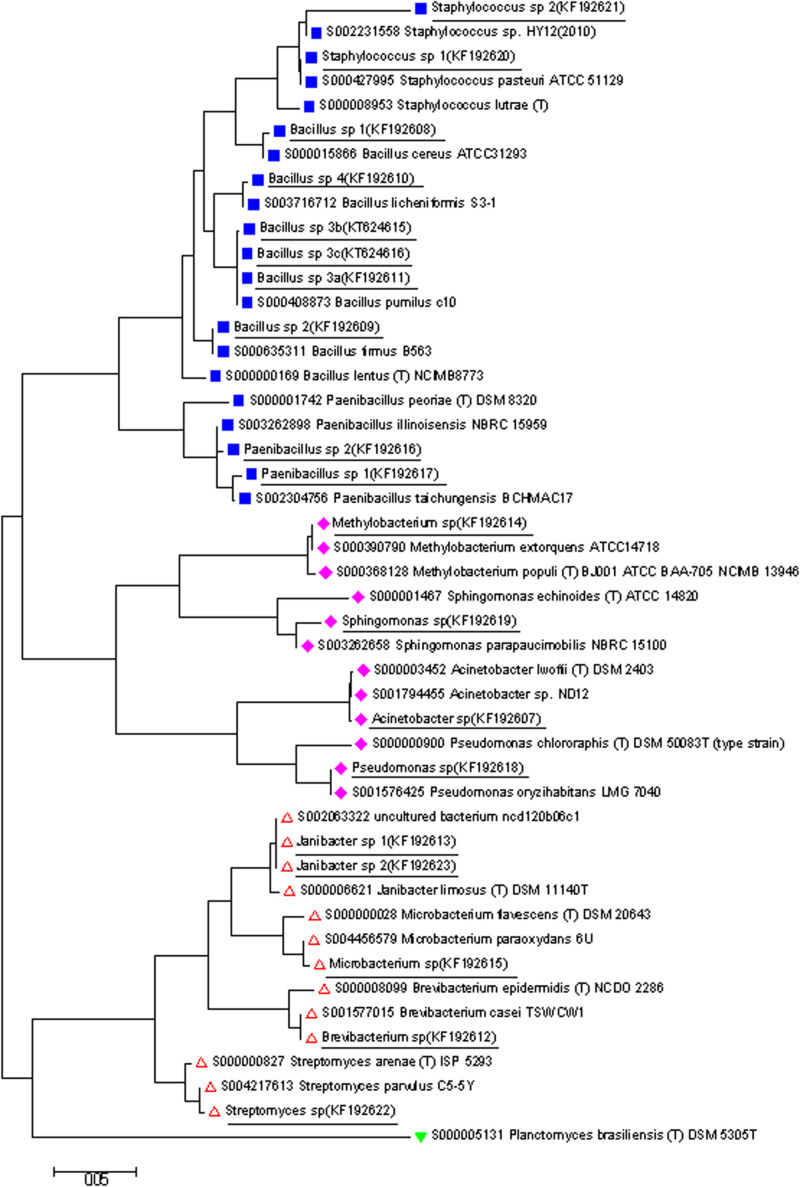
A phylogenetic tree based on the 16S rDNA gene sequences from isolates determined in this study (shown underlined), and closely related 16S sequences from isolates available from RDP database. The tree was generated by Neighbor Joining method in Mega. Phylum groups: 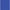 Firmicutes, 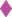 Proteobacteria, 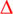 Actinobacteria, and 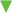 Planctomycetes (out group).

Based on phylogenetic analyses, the strains were placed into three phyla: Actinobacteria (5), Proteobacteria (4), and Firmicutes (10) (Figure 3).

### Design, Testing and Use of Specific Primers

Between 2 and 5 primer pairs were designed for specific amplification of each OTU. None of the primer pairs amplified DNA from non-contaminated plants (data not shown) and at least one primer pair amplified DNA for each OTU (Table 3) showing strong and specific amplification with their corresponding bacteria for: *Sphingomonas* spp., *Bacillus* spp_1 and *Bacillus* spp_3, *Paenibacillus* spp_1 and *Paenibacillus* spp_2, *Methylobacterium* spp., *Brevibacterium* spp., *Acinetobacter* spp., *Microbacterium* spp., and *Streptomyces* spp. In the case of *Bacillus* spp_3 no attempt was made to design primers for the closely related OTUs a, b, and c and they thus all had an amplified product using the same primer pair.

**TABLE 3:** Selected primers for the specific PCR amplification of bacterial 16S rDNA genes of different taxonomic groups.

**Primer name^a^**	**Sequence (5′–3′)**	**Expected approximate product size (bp)**	***T*_a_^b^ (°C)**
*Sphingomonas*_F2	TACCGGATGATGACGAATGTCC	539	47.5
*Sphingomonas*_R1	ATACCAGTCCAGTCAGCCGCC		
*Staphylococcus_*1_F2	GGTTCAATAGTGAAAGACGGCCTTGC	807	47
*Staphylococcus_*1_R1	CTCAAGATTTGGTAAGGTT		
*Bacillus*_1_F3	GCGGCTTCGGCTGTCACTTATG	657	61
*Bacillus*_1_R1	GGTTTCCGCCCTTTAGTGCTGA		
*Paenibacillus*_1_F2	CGCTTGGGAGAGTAACTGCTCTC	376	58
*Paenibacillus_1*_R1	CTAGCACTCATCGTTTACGG		
*Methylobacterium*_F2	GGAATAACTCAGGGAAACTT	668	52
*Methylobacterium*_R1	CTGACCTGCAAGCAGGCCAACGG		
*Bacillus_*2*_*F1	AAAGCTGAAAGATGGTTTCGGCTA	667	59
*Bacillus_*2*_*R1	GCAGCACTAAAGGGCGGAAA		
*Bacillus_*3*_*F2*	ATGGTTCAAGGATGAAAGACGGTTT	464	43
*Bacillus_*3*_*R2*	TTTCCCAGTTTCCAATGACC		
*Brevibacterium_*F3	GTCTAATACCGGATACGACTG	668	55
*Brevibacterium_*R1	GAGAACGTGGAATGCCC		
*Bacillus_*4*_*F1	GTAAGGTTCTTCGCGTTGCTTCG	857	63
*Bacillus*_4*_*R2	AGACTGGGATAACTCCGGGAAA		
*Janibacter*_F3	CCCAGACTCTGGAATAAGCGC	855	58
*Janibacter*_R4	TTTCCGGTATATGTCAAGCC		
*Paenibacillus_*2*_*F1	GCTTTCTTCGCCTGAAGGAAG	712	65
*Paenibacillus_*2*_*R1	CCCAGGCGGAATGCTTAATGTGT		
*Acinetobacter*_F3	AGCTGCGCCACTAAAGCCTC	760	61
*Acinetobacter*_R1	CCACTAATAGGCAGATTCCTAAGCA		
*Microbacterium*_F1	GGCGTCTAATACTGGATATGTGACGTG	625	62.5
*Microbacterium*_R1	TCACGGAATCCGTGGAAAGGA		
*Janibacter*_2_F2	ACACTCTAGTCTGCCCGTACCCAC	488	65
*Janibacter*_2_R2	GCGCTGGAAACGGCGTCTAATACT		
*Streptomyces*_F1	ACGGACAACGTGGAATGTTG	656	60
*Streptomyces*_R2	TCACAGATGCCCGTGAAGGTCA		

^*a*^Primer abbreviations: F, forward; R, reverse; ^*b*^T_*a*_, annealing temperature. *amplified Bacillus sp.3 a, b, and c (Table 2).

Using the newly designed primers, 180 of the originally isolated bacteria (isolates from CIP 421133, 400514, 400256, and 442768 were lost) were tested and re-grouped according to the PCR results (Table 4). Most of the 180 isolates belonged to two majority groups, i.e., 74 *Bacillus* spp. and 80 *Paenibacillus* spp. One isolate each from group “A” and “B” were not amplified by any of the specific primers and their 16S sequences were therefore determined separately identifying them as OTUs related to *Pseudomonas oryzihabitans* and *Staphylococcus pasteuri* (see previous section). PCR fragments of the three most representative OTUs were chosen for sequence confirmation by randomly sequencing 3 isolates from *Bacillus* spp.1, 6 isolates from *Bacillus* spp.3, and 7 isolates from *Paenibacillus* spp.1 (Supplementary Table S2). In all cases the nucleotide sequences confirmed specific amplification showing 99–100% nt identity to their corresponding 16S rDNA sequence.

**TABLE 4:** Final classification of 180 bacteria isolated from *in vitro* cultures of *Ipomoea batatas.*

**Morphological group**	**No. isolates identified morphologically**	**OTU**	**No. identified by PCR**	**% strains total by molecular detection**
A	2	*Sphingomonas* sp	1	0.56
	0	*Pseudomonas* sp.	1	0.56
B	8	*Staphylococcus* sp.1	7	3.89
	0	*Staphylococcus* sp.2	1	0.56
C	29	*Bacillus* sp.1	40	22.22
D	82	*Paenibacillus* sp.1	75	41.67
E	1	*Methylobacterium* sp.	1	0.56
F	1	*Bacillus* sp.2	1	0.56
G	1	*Brevibacterium* sp.	1	0.56
H,I,J	39	*Bacillus* sp.3a, b, c*	30	16.67
K	1	*Bacillus* sp.4	3	1.67
L	7	*Janibacter* sp.	7	3.89
M	2	*Paenibacillus* sp.2	5	2.78
N	1	*Microbacterium* sp.	1	0.56
O	1	*Acinetobacter* sp.	1	0.56
P	4	*Janibacter* sp.2	4	2.22
Q	1	*Streptomyces* sp.	1	0.56
**Total**				100

*^∗^Bacillus sp.3 a, b, and c amplified with Bacillus_3_F2 and Bacillus_3_R2 primer pair (Table 2).*

Table 4 shows the grouping based on identification by PCR whereas Supplementary Table S2 shows the sweetpotato accession numbers from which these bacteria were isolated.

### Sensitivity of Detection

The detection limits of each primer pair are shown in Table 5 according to the method of extracting the DNA. The detection limit of each primer pair, amplifying directly from the bacterial suspension or from DNA extracted from contaminated plants for the 14 bacterial species ranged from 5 to 3.33 × 10^7^ and from 50 to 3.33 × 10^9^ cfu ml^–1^, respectively. Only the primer pair for *Janibacter* sp. 1, was unable to detect the bacteria directly even at the highest concentration of 10^9^ cfu ml^–1^. On the other hand, attempts to design a generic primer pair [pMF1 (5′- GGC GTC TAA TAC TGG ATA TGT GAC GTG -3′) and pMR (5′-GGA CTA CCA GGG TAT CTA A-3′)] to detect all bacterial groups identified in this study was unsuccessful (data not shown).

**TABLE 5:** Sensitivity of primers for detecting each bacterial group (OTU).

**OTU**	**Primer pairs^a^**	**Detection limit directly from bacterial culture (cfu ml**^–^**^1^)**	**Detection limit of DNA extracted from contaminated plants (cfu ml**^–^**^1^)**
*Sphingomonas* sp	F2R1	5 × 10^1^	5 × 10^1^
*Staphylococcus* sp.1	F2R1	4.55 × 10^4^	4.55 × 10^6^
*Bacillus* sp.1	F3R1	3.13 × 10^3^	3.13 × 10^1^
*Paenibacillus* sp.1	F2R1	5	5 × 10^2^
*Methylobacterium* sp.	F2R1	4.55 × 10^5^	4.55 × 10^8^
*Bacillus* sp.2	F1R1	5 × 10^2^	5 × 10^3^
*Bacillus* sp.3 a, b, c	F2R2	5 × 10^1^	5 × 10^1^
*Brevibacterium* sp.	F3R1	4.76 × 10^3^	4.76 × 10^5^
*Bacillus* sp.4	F1R2	4.55 × 10^4^	4.55 × 10^2^
*Janibacter* sp. 1	F3R4	ND	ND
*Paenibacillus* sp.2	F1R1	4.35 × 10^1^	4.35 × 10^5^
*Acinetobacter* sp.	F3R1	5 × 10^2^	5 × 10^6^
*Microbacterium* sp.	F1R1	4.55 × 10^5^	4.55 × 10^9^
*Janibacter* sp.2	F2R2	3.33 × 10^7^	3.33 × 10^9^
*Streptomyces* sp.	F1R2	1.67 × 10^3^	1.67 × 10^4^

^*a*^Primer abbreviations: F, forward; R, reverse. ND, not detected in the range 2.86 × 10^9^–2.86 × *10^0^.

### Consistency Between Molecular and Morphological Groupings

Statistical analysis using a test of proportions was used for determining consistency between isolate identification by PCR and classical methods using the 180 isolates for which both tests had been performed. Due to the differences between the morphological and 16S based groupings in the OTUs of *Paenibacillus* sp. 1 and *Bacillus* sp. 3, the hypothesis that there was no significant difference was rejected with a *p*-value of 0.021 and 0.005, respectively (α = 0.05). The margin of error of the conventional grouping of 180 isolations as compared to the PCR based identification was 10% in total.

### Elimination of Bacteria in the *in vitro* Collection

Of the 240 accessions confirmed to be contaminated with bacteria, all were processed for bacterial elimination. To date, 197 accessions have been reintroduced, but 6 of them died. The surviving 191 accessions were all processed using the method described above. PCR testing of the *in vitro* accessions processed for bacterial elimination (*n* = 125) returned negative results and plants have not shown evidence of bacterial contamination ever since their cleanup in 2012 (Supplementary Table S2), suggesting the plants were truly free of bacterial contamination and that the PCR results were accurate in that respect. The length of time required to clean each accession was 14 months. The cleaned accessions were successfully established in CIP’s *in vitro* germplasm collection.

The accessions that died (6) were weak and showed poor growth *in vitro* even with monthly subcultures with both MPB liquid medium and the same supplemented with coconut water 10 ml/l (sterilized by filtration and added after autoclaving). It is unknown whether this -low or null growth is an effect of the anti-bacterial treatment, a genotypic effect, a reaction to the -disinfection treatment or any other cause. Whatever the reason, these accessions represented only 2.5% of those treated and they will be retreated to obtain new plant material for re-isolation into *in vitro*.

## Discussion

This study was initiated to characterize and identity bacteria contaminating sweetpotato tissue cultures by partial 16S rDNA sequencing, as well as to develop PCR based diagnostic tests for the rapid and sensitive detection of contaminants directly from tissue culture plants without the need of performing microbial sub-cultures. This is critical because CIP is the custodian of the world’s largest *in vitro* genebank which maintains the global *in trust* clonal collection of 4,734 accessions of potato, 5,573 accessions of sweetpotato and 1,561 accessions of other Andean root and tuber crops (data as of January 2020).

Studies of sweetpotato bacterial contaminants has previously only been performed at a morphological level which identified *Corynebacterium*, *Klebsiella*, and *Pseudomonas* spp. (Jena and Samal, 2011), *Enterobacter*, *Rahnella*, *Rhodanobacter*, *Stenotrophomonas*, *Xanthomonas*, and *Phyllobacterium* (Khan and Doty, 2009). Here, through morphological characterization and partial 16S rDNA sequencing, we were able to detect at least 19 OTUs of bacteria isolated from the *in vitro* cultures of sweetpotato (Tables 1, 2). The identities as determined by 16S rDNA sequences were in agreement with the published morphological and biochemical characteristics of closely related bacteria, in all isolates (Bourque et al., 1992; Gruner et al., 1994; Doukyu et al., 2003; Laffineur et al., 2003; Constantiniu et al., 2004; Li et al., 2004; Collins, 2006; Lee et al., 2008; Hendricks and Boone, 2009; Savini et al., 2009; Shivaji et al., 2009; Usha, 2011). Although the results from comparisons to the RDP and NCBI databases were very similar, they were not always identical (Table 2). Whereas the NCBI database is far larger, providing the opportunity to compare results against more samples, the exact identity of the deposited sequences is not curated as in the case of the RDP database. Considering this, for this study, the identity of entries in the RDP database are considered more reliable (Cole et al., 2011). Nevertheless, since we have only used a partial 16S rDNA sequence in our analysis and different bacterial species can have as high as 97% sequence similarity over the entire 16S rDNA gene, we do not consider this study an unambiguous identification of each isolate to the species level. Thus, although high sequence identity can certainly identify an isolate as closely related to the corresponding species, we have considered them as OTUs of an unverified species within a genus level. We have distinguished related OTUs with different numbers, and additionally letters when they were very similar (Table 2).

The designing of specific primers based on 16S rDNA sequences for the identification of each individual isolate (Table 3), enabled the rapid confirmation of the identity of the remaining isolates and also to identify new OTUs that were not amplified by any of the designed primers (i.e., in morphological groups A and B). The specificity of the primers was confirmed by sequencing the amplified fragments from randomly selected isolates. *Bacillus* spp. and *Paenibacillus* spp., often found in the soil (Supplementary Table S3), represented the majority of bacteria identified in this research (Table 4), possibly because they can live endogenously within the plant or root as a result of colonization through physical damage, during growth or as an escape of initial sterilization during tissue culture isolation (Isenegger et al., 2003). Indeed, there are reports of autoclave and alcohol resistant, spore-forming bacteria (Thomas and Soly, 2009; Thomas et al., 2009; Thomas and Aswath, 2014), and thus this cannot be ruled out. Yet, since bacterial contamination is relatively common in sweetpotato tissue cultures (10% of cultures from this study), but is rarely seen in the *in vitro* cultures of the other eight crops maintained *in vitro* at CIP, and appears to be independent of the geographic origin of the sweetpotato accessions (data not shown), the source of these *in vitro* contaminating bacteria is likely endogenous from the original sweetpotato isolates.

In addition to being very specific, some primers also proved to be extremely sensitive, able to detect as little as 31–50 cfu ml^–1^, in the case of *Sphingomonas* spp. and *Bacillus* spp.1 (Table 5), in mixes with plant material. The detection limit of primers for *Bacillus* spp.3 (closely related to *B. pumilus*) primers and for *Bacillus* spp.1 (closely related to *B. cereus*) were 20 and almost 5 × 10^3^ fold more sensitive, respectively, than those previously reported (Isenegger et al., 2003; Kałużna et al., 2014). Only primers for *Janibacter* spp.1 were not sensitive enough to detect the bacteria either directly from culture (without DNA extraction), or from contaminated plants (with DNA extraction).

Whereas available universal primers for amplification of 16S rDNA such as pA and pF are very useful for amplifying 16S rDNA from purified cultures, they are not useful for detecting bacterial contaminants in plants as they also amplify plant rDNA. It would be extremely useful to have highly specific primers that could amplify all endophytic bacterial but that would not amplify plant DNA (Sun et al., 2008). To this end, we tested several previously reported primer pairs resulting in no amplification [primer pairs 27F and 904R, 27F and 1185mR, 895F and 1492R (Hodkinson and Lutzoni, 2009)], or only amplification with some of the isolates [primer pair 799f and 1492r (Sun et al., 2008) only could amplify *Paenibacillus* sp. and *Acinetobacter* sp.]. Thus, attempts to design our own generic primers were also unsuccessful, either being unspecific, or lacking sufficient sensitivity. Future work to design degenerated primers with a broader specificity for different bacteria would be valuable to facilitate their rapid detection.

The habitats of the identified bacteria are mostly ubiquitous (Supplementary Table S3), having previously been associated with diverse plant cultures or soil, and it is difficult to say definitively if they originated from environmental contamination during tissue culture initiation, or represent endophytic bacteria that were present in the plants prior to introduction in tissue culture. Nevertheless, some of these bacteria have been reported to have beneficial effects on plant growth (Supplementary Table S3) and may represent a valuable source of potential plant growth promoting or otherwise beneficial bacteria that can be explored and/or exploited in the future. There is no question that some bacteria can be beneficial to certain crops when associated with them such as the co-existence of some microbes have been described to promote plant growth in crops (Ferreira et al., 2019) or in the case of *Acinetobacter* spp., has been found to produce the auxin indole acetic acid (Rokhbakhsh-Zamin et al., 2011). Beneficial effects, however, may not be evident *in vitro* where conditions are already enhanced for shoot growth. Experimentally determining whether these bacteria are beneficial to sweetpotato plants in the field would be very interesting, yet challenging, as the beneficial effect may be site or environment specific. Unfortunately, answering this question was beyond the scope of this experiment, however, the hope is that this study provides tools that could be used in the future to better elucidate the role of beneficial bacteria in plants.

In the context of plant tissue culture, any foreign organism growing in the culture is considered a contaminant whether from an endogenous or and exogenous source. This is especially true in the case of material destined for international distribution, such as the CIP collection, as phytosanitary certification is needed and the recipient may also maintain the material long-term *in vitro* where absolute sterility of the culture environment is required. Additionally, experience at CIP shows most contaminant bacteria affect the viability of *in vitro* cultures negatively. During sweetpotato *in vitro* collection maintenance, accessions with signs of bacterial contaminations similar to those of the most frequent species found in this study (*P. taichungensis*, *B. cereus*, and *B. pumilus*) grew slowly and produced weak explants that more frequently died during subculture. Thus, elimination of bacterial contaminants is a fundamental activity to guarantee conservation and secure exchange of germplasm, and like pathogen elimination is an essential procedure applied in CIP’s Genebank.

While the long-term use of antibiotics in tissue culture is feasible, the CIP genebank takes all precautions to avoid the long-term use of agents such as antibiotics as it is unknown what long-term effects this may have with *in vitro* plants or the evolution of resistant bacteria. Also, antibiotics are not always fully effective and bacteria can remain present latently in plant tissue, re-flourishing when conditions are appropriate. CIP manages infected germplasm in specific facilities with specialized methods and staff experienced in bacterial elimination from plant tissue cultures. Since detection through visual inspection and/or bacteria culture tests using NB are not reliable there was the need to develop more sensitive detection systems based on molecular tools.

The PCR primers and their use in detection of bacterial contaminants enhances CIP’s quality control practices and helps ensure the inadvertent transfer of the contaminants to healthy tissues in the tissue culture process as well as when distributing germplasm. The cleaning method described is relatively simple, but is lengthy and requires a greenhouse step followed by re-isolation into *in vitro*. Despite this, the bacterial cleaning procedure works extremely well with 100% of treated accessions resulting bacterial-free.

In summary, this research identified a wide diversity of bacterial species associated with the *in vitro* cultures of *Ipomoea batatas*, and developed a rapid PCR detection method that can be applied directly to tissue cultured plants without the need to isolate bacteria microbiologically first. The primers developed in this study provide potential tools for tissue culture facilities to rapidly screen the *in vitro* materials for undetected bacteria. In addition, this study also provides a highly efficient method for the elimination of putative endogenous bacteria from plants enabling the establishment of bacterial-free *in vitro* cultures.

## Data Availability Statement

The datasets presented in this study can be found in online repositories. The names of the repository/repositories and accession number(s) can be found below: https://www.ncbi.nlm. nih.gov/, KF192607; https://www.ncbi.nlm.nih.gov/, KF192608; https://www.ncbi.nlm.nih.gov/, KF192609; https://www.ncbi. nlm.nih.gov/, KF192610; https://www.ncbi.nlm.nih.gov/, KF19 2611; https://www.ncbi.nlm.nih.gov/, KF192612; https://www. ncbi.nlm.nih.gov/, KF192613; https://www.ncbi.nlm.nih.gov/, KF192614; https://www.ncbi.nlm.nih.gov/, KF192615; https://www.ncbi.nlm.nih.gov/, KF192616; https://www.ncbi.nlm.nih. gov/, KF192617; https://www.ncbi.nlm.nih.gov/, KF192619; https://www.ncbi.nlm.nih.gov/, KF192620; https://www.ncbi.nlm.nih.gov/, KF192622; https://www.ncbi.nlm.nih.gov/, KF19 2623; https://www.ncbi.nlm.nih.gov/, KF192618; https://www.ncbi.nlm.nih.gov/, KF192621; https://www.ncbi.nlm.nih.gov/, KF192616; https://www.ncbi.nlm.nih.gov/, KF192615.

## Author Contributions

JK, AP, and LG designed the experiments. MI, CM, JC, and CR performed the experiments. MI and JK analyzed the data and wrote the manuscript with contributions from all authors.

## Conflict of Interest

The authors declare that the research was conducted in the absence of any commercial or financial relationships that could be construed as a potential conflict of interest.

## Footnotes

1 http://www.ncbi.nlm.nih.gov/

2 http://rdp.cme.msu.edu/

## Supplementary Material

TABLE S1 Accession numbers of sweetpotato plants from which 184 bacteria corresponding to different morphological groups were isolated.

TABLE S2 Accession numbers of *in vitro* sweetpotato plants and the identity of bacteria isolated from them, based on 16S sequence.

TABLE S3 Reported habitat and benefits of bacteria related to those identified in this study.
