# Supplementary material for: Identification and Control of Latent Bacteria in in vitro Cultures of Sweetpotato [Ipomoea batatas (L.) Lam]
Source: Front Plant Sci. 2020 Jul 3;11:903. doi: 10.3389/fpls.2020.00903 (PMC7350948; doi:10.3389/fpls.2020.00903)
Supplement: TABLE S1 — Accession numbers of sweetpotato plants from which 184 bacteria corresponding to different morphological groups were isolated. [file Data_Sheet_1.PDF]

Table S1. Accession numbers of sweetpotato plants from which 184 bacteria corresponding to different morphological groups were isolated.

Morphologic group	Nº CIP	Nº
**A**	420278 - 420933	2
**B**	400311 - 421103 - 440699 – 401538 – 421034 – 420285 – 400270 - 422567	8
**C**	421426- 400074 - 402751 - 440157 - 422503 - 422505 - 401030- 401031- 400256 - 400390- 441259 - 440765 - 441159 - 440801 - 441168 -440473- 440771 - 441017 - 401320 - 401322 - 402897 - 402915 - 403009 -403021- 420538 - 422060- 440189 - 440142- 440266	29
**D**	420251- 420086 - 420341- 421135- 420345- 420353 - 422557- 401014 - 422540- 422556 - 422558- 421136 - 422584- 400359 - 400371 - 440026- 422642 - 420246 - 441715- 441787- 400291 - 400131- 400154 -400148- 400189- 400213- 400277- 400399- 400426- 400838- 401522- 400317- 400182 - 400308- 400274 - 400848 - 400874- 400982- 401042- 401043 - 401211- 400609 - 420580 - 420619-420177- 420830- 420456- 442928- 442802- 442198 - 442379 - 442797- 442748– 441580 - 441609 - 440922 - 441615 - 441036 - 441624- 441159 - 440712-440717- 440792- 441422- 441194- 441177- 402789 - 440008- 440309 - 440347- 188005.1- 440188 - 440236- 199014.2 - 189148.65- 400293 - 400895 - 400997- 400192 - 441473 - 440762 - 440473 - 400514 - 400256 - 442768	85
**E**	400108	1
**F**	442368	1
**G**	442536	1
**H**	420613 - 400902 - 401197 - 420760 - 440214 - 440844 - 441280 - 401326 - 440099 - 421593 - 402749 - 440786	12
**I**	442507 - 400106 - 400483 - 440326	4
**J**	402715 - 421092 - 421099 - 422534 - 441548- 441777 - 401084 - 400090 - 401400 - 420603 - 442775 - 440616 - 430394 - 401533 - 403043 - 440023 - 440141 - 440298 - 400441 - 401549 - 422556 - 421135 - 420326	23
**K**	441180	1
**L**	400062 - 420621 - 440699 - 441516 - 440473 - 440314 - 441752	7
**M**	400280 - 442616	2
**N**	440199	1
**O**	421115 - 421133	2
**P**	187002.1 - 441711 - 400547 - 440020	4
**Q**	420405	1
