# Supplementary material for: Identification and Control of Latent Bacteria in in vitro Cultures of Sweetpotato [Ipomoea batatas (L.) Lam]
Source: Front Plant Sci. 2020 Jul 3;11:903. doi: 10.3389/fpls.2020.00903 (PMC7350948; doi:10.3389/fpls.2020.00903)
Supplement: TABLE S2 — Accession numbers of in vitro sweetpotato plants and the identity of bacteria isolated from them, based on 16S sequence. [file Data_Sheet_2.PDF]

Table S2. Accession numbers of 180 *in- vitro* sweetpotato plants and the identity of bacteria isolated from them, based on 16S sequence.

NºCIP [*]	Genera of bacterium identified [**]
420278[a,f] (L)	*Sphingomonas* sp.
400311[a,f] (S) -421103[e,f] (L) – 440699[e] (L)– 401538[e,f] (S) – 421034[e,f] (L) – 420285[e,f] (S)– 400270[e,f] (L)	*Staphylococcus* sp. 1
421426[a] (L) - 441280[b,f] (L) - 440844[b,f] (L) -420760[b,f] (L) - 421593[b,f] (L) - 440786[b,f] (L) - 440099[b,f] (L) - 440214[b] (L) - 401326[c,f] (L) - 402749[c,f] (L) - 400192[c] (L) - 400895[c] (L) - 400074[d](L) - 402751[d] (L) - 440157[d] (L)- 422503[e] (L) - 422505[e](L) - 401030[e,f] (L) - 401031[e] (L) - 400256[e,f] (L) - 400390[e] (L) - 441259[e,f] (L) - 440765[e,f] (L) - 441159[e,f] (L) -440801[e,f] (L) - 441168[e,f] (L) - 440473[e,f] (L) - 440771[e,f] (L) - 441017[e,f] (L) - 401320[e] (L) - 401322[e,f] (L) - 402897[e,f] (L) - 402915[e] (L) - 403009[e,f] (L) - 403021[e] (L) - 420538[e,f] (L) –422060[e,f] (L) – 440189[e,f] (L) – 440142[e,f] (L) - 440266[e,f] (L)	*Bacillus* sp. 1
420251[a] (S) - 420086[d,f] (L) - 420341[d,f] (L) - 421135[d,f] (L) - 420345[d,f] (L) - 420353[d,f] (L) -422557[d,f] (L) - 401014[d,f] (L) - 422540[e,f] (L) - 422556[e] (L) - 422558[e,f] (L) - 421136[e,f] (S) - 422584[e] (S)- 400359[e,f] (L) - 400371[e] (L) - 440026[e,f] (L) - 422642[e,f] (L) - 420246[e,f] (S) - 441715[e,f] (L) - 441787[e] (L) - 400291[e,f] (L) - 400131[e,f] (L) - 400154[e] (L) - 400148[e,f] (L) - 400189[e,f] (L) - 400213[e,f] (S) - 400277[e] (S) - 400399[e] (L) - 400426[e,f] (L) - 400838[e,f] (L) - 401522[e] (S) - 400317[e,f](L) - 400182[e] (L) - 400308[e] (S) - 400274[e,f] (L) - 400848[e,f] (S) - 400874[e] (L) - 400982[e] (S) - 401042[e] (S) - 401043[e](S)- 401211[e,f] (L) - 400609[e] (L) - 420580[e,f] (S) - 420619[e,f] (S) - 420177[e] (S) - 420830[e,f] (S) - 420456[e] (S) - 442928[e,f] (S) - 442802[e,f] (S) -442198[e] (L)- 442379[e,f] (L) - 442797[e,f] (S) - 442748[e,f] (L) - 441580[e,f] (S) - 441609[e,f] (L) - 440922[e,f] (S) - 441615[e,f] (L) - 441036[e,f] (L) - 441624[e,f] (S) - 441159[e] (L)- 440712[e,f] (L) - 440717[e,f] (L) - 440792[e,f] (L) - 441422[e,f] (L) - 441194[e,f] (L) - 441177[e,f] (L) - 402789[e,f] (S) - 440008[e,f] (S) - 440309[e,f] (L) - 440347[e,f] (S)- 188005.1[e,f] (S) - 440188[e,f] (L) - 440236[e,f] (L) - 199014.2[e,f] (L) - 189148.65[f] (S)	*Paenibacillus* sp. 1
400108 [a,f] (L)	*Methylobacterium* sp.
442368 [a,f] (L)	*Bacillus* sp. 2
442536 [a,f] (L)	*Brevibacterium* sp.
420613[a] (L) - 442507[a] (L) - 402715[a,f] (S) - 440326[d,f] (S) - 420326[d,f] (L) - 422556[d] (L) - 421135[d] (L) - 401549[d] (L) - 400441[d,f] (L) - 400902[e,f] (L) -401197[e,f] (L) - 400106[e,f] (L) -400483[e,f] (L) - 421092[e] (L) -421099[e,f] (L) - 422534[e,f] (L) - 441548[e,f] (L) - 441777[e,f] (L) - 401084[e,f] (L) - 400090[e,f] (L) - 401400[e,f] (L) - 420603[e,f] (S) - 442775[e,f] (L) - 440616[e,f] (L) - 430394[e,f] (L) - 401533[e] (L) - 403043[e,f] (L) - 440023[e,f] (L) - 440141[e] (L) - 440298[e] (L)	*Bacillus* sp. 3a,b,c.
441180[a,f] (L) – 440473[b] (L) -400293[c,f] (L)	*Bacillus* sp. 4
400062[a,f] (S) – 420621 [e,f] (L) –440699 [e] (L) - 441516 [e,f] (L) - 440473 [e] (L) - 440314 [e,f] (L) - 441752 [e,f] (L)	*Janibacter* sp. 1
400280[a,f] (S) - 440762 [b,f] (L) - 400997 [c] (L)– 441473 [c,f] (L) - 442616 [e,f] (S)	*Paenibacillus* sp. 2
440199[a] (L)	*Microbacterium* sp.
421115[a,f] (L)	*Acinetobacter* sp.
187002.1[a] (L)-400547 [d] (L) – 440020 [d] (L) - 441711 [e] (L)	*Janibacter* sp 2.
420405[a,f] (S)	*Streptomyces* sp. 1
422567[a] (L)	*Staphylococcus* sp. 2
420933[a] (L)	*Pseudomonas* sp.

[*] Detection Medium: Liquid (L), Solid (S).[a]First sequenced isolate used for design primers, [b]Regrouped isolates based on PCR with specific primer and sequencing, [c]Regrouped isolates based on PCR with specific primer, [d]Isolates chosen randomly to confirm PCR based grouping by sequencing, [e]Isolates based on PCR with specific primers and [f]rechecked experiment with the *in vitro* plants that were reintroduced after the Dimanin® treatment.

[**] *Bacillus* sp.3 a, b and c amplified with *Bacillus*_3_F2 and *Bacillus*_3_R2 primer pair (Table 2).
