# Supplementary material for: Identification and Control of Latent Bacteria in in vitro Cultures of Sweetpotato [Ipomoea batatas (L.) Lam]
Source: Front Plant Sci. 2020 Jul 3;11:903. doi: 10.3389/fpls.2020.00903 (PMC7350948; doi:10.3389/fpls.2020.00903)
Supplement: TABLE S3 — Reported habitat and benefits of bacteria related to those identified in this study. [file Data_Sheet_3.pdf]

Table S3. Reported habitat and benefits of bacteria related to those identified in this study

Bacterium	Reported habitats	Reported in plant culture	Reported benefits
Sphingomonas sp.	Different aqueous and terrestrial habitats, as well as from plant root systems, clinical specimens, and other sources (Balkwill et al., 2006).	Papaya (*In vitro*) (Thomas et al., 2007)	Can prevent the proliferation of invasive bacterial pathogens. (Innerebner et al., 2011)
Staphylococcus pasteuri	Humans, animals and food samples. (Chesneau et al., 1993)	Orchids and roses, among others (*In vitro*) (Zawadzka et al., 2009)	
Bacillus cereus	Soil, litter, sediments and plants. (Mandic-Mulec and Prosser, 2011)	Cassava, kenaf and banana (*In vitro*) (Odutayo et al., 2007)	Can inhibit diseases caused by pathogenic protozoa and also enhance the growth of plants (Jensen et al., 2003). It naturally produces antibiotics zwittermicin A and Kanosamine which can inhibit the growth of plant pathogens, certain fungi and some bacteria. (Silo-Suh et al., 1994)
Paenibacillus sp.	Soil and in the rhizosphere. (McSpadden Gardener, 2004)	Banana (*In vitro*) (Thomas and Soly, 2009)	Promote plant growth and suppress pests. (McSpadden Gardener, 2004)
Methylobacterium sp.	Widely distributed, colonizing various aquatic and terrestrial ecosystems, such as lakes, rivers and marine systems, groundwater, human skin, air, and are common in soils and surfaces of leaves and other plants parts. (Lidstrom and Chistoserdova, 2002;Schmidt, 2010)	Papaya (Thomas et al., 2007) and Chrysanthemum (Thomas et al., 2009) (*In vitro)*	Produce plant hormones such as cytokinins and auxins, and vitamin B12 which stimulate plant growth. (Abanda-Nkpwatt et al., 2006)
Bacillus firmus	Isolated from manure. (Berić et al., 2009). Also, live in forest soil and environments where their spores survive high temperatures, such as buried ashes buried. (Mandic-Mulec and Prosser, 2011)	Eucalyptus (Greenhouse) (Díaz et al., 2009)	Reported as promoters of plant growth. (Khan and Patel, 2007)
Brevibacterium sp.	Found in the soil (Fernández-Luqueño et al., 2011), skin and blood. (Winn et al., 2006)	Banana (*In vitro*) (Thomas et al., 2008)	
Bacillus pumilus	Coastal and marine environments, is highly resistant to extreme environmental conditions such as non-availability of nutrients, drying, irradiation H_2O_2, and chemical disinfection. (Berić et al., 2009). Often found in soil. (Isenegger et al., 2003)	Banana (Thomas et al., 2009), Grapes (Díaz et al., 2009), Potato (Isenegger et al., 2003) (*In vitro*)	Reported as promoters of plant growth. (Lin et al., 2012)
Microbacterium sp.	Isolated from the environment (Funke et al., 1997) as well as human clinical specimens. (Gneiding et al., 2008)	Papaya (*In vitro*) (Thomas et al., 2007)	Are able to fix N_2 for the plant. (Lin et al., 2012)

Acinetobacter sp.	Their natural habitats are the soil and water and have been isolated from food, arthropods and the environment. In humans it may colonize skin, wounds, respiratory and gastrointestinal tracts. (Munoz-Price and Weinstein, 2008)	Banana (*In vitro*) (Thomas and Soly, 2009)	Reported to promote plant growth. (Rokhbakhsh-Zamin et al., 2011)
Streptomyces sp.	Widely distributed in soil, water and colonizing plants. (Usha et al., 2010) (Usha, 2011).	Sweet potato (Field) (Zhang et al., 2003)	Are known to antagonize plant pathogens (Zahar Haichar et al., 2008), whereas others are considered pathogens. (Zhang et al., 2003)

Abanda-Nkpwatt, D., Müsch, M., Tschiersch, J., Boettner, M., and Schwab, W. (2006). Molecular interaction between *Methylobacterium extorquens* and seedlings: growth promotion, methanol consumption, and localization of the methanol emission site. *Journal of experimental botany* 57**,** 4025-4032.

Balkwill, D.L., Fredrickson, J.K., and Romine, M.F. (2006). "*Sphingomonas* and related genera," in *The prokaryotes,* eds. M. Dworkin, S. Falkow, E. Rosenberg, K. Schleifer & E. Stackebrandt. (New York: Springer), 605-629.

Berić, T., Urdaci, M.C., Stanković, S., and Knežević-Vukčević, J. (2009). RAPD analysis of genetic diversity and qualitative assessment of hydrolytic activities in a collection of *Bacillus* sp. isolate. *Archives of Biological Sciences* 61**,** 645-652.

Chesneau, O., Morvan, A., Grimont, F., Labischinski, H., and El Solh, N. (1993). *Staphylococcus pasteuri* sp. nov., isolated from human, animal, and food specimens. *International journal of systematic bacteriology* 43**,** 237-244.

Díaz, K., Valiente, C., Martínez, M., Castillo, M., and Sanfuentes, E. (2009). Root-promoting rhizobacteria in *Eucalyptus globulus* cuttings. *World Journal of Microbiology and Biotechnology* 25**,** 867-873.

Fernández-Luqueño, F., Valenzuela-Encinas, C., Marsch, R., Martínez-Suárez, C., Vázquez-Núñez, E., and Dendooven, L. (2011). Microbial communities to mitigate contamination of PAHs in soil—possibilities and challenges: a review. *Environmental Science and Pollution Research* 18**,** 12-30.

Funke, G., Haase, G., Schnitzler, N., Schrage, N., and Reinert, R.R. (1997). Endophthalmitis due to *Microbacterium* species: case report and review of *Microbacterium* infections. *Clinical infectious diseases* 24**,** 713-716.

Gneiding, K., Frodl, R., and Funke, G. (2008). Identities of *Microbacterium* spp. encountered in human clinical specimens. *Journal of clinical microbiology* 46**,** 3646-3652.

Innerebner, G., Knief, C., and Vorholt, J.A. (2011). Protection of *Arabidopsis thaliana* against leaf-pathogenic *Pseudomonas syringae* by *Sphingomonas* strains in a controlled model system. *Applied and environmental microbiology* 77**,** 3202-3210.

Isenegger, D., Taylor, P., Mullins, K., Mcgregor, G., Barlass, M., and Hutchinson, J. (2003). Molecular detection of a bacterial contaminant *Bacillus pumilus* in symptomless potato plant tissue cultures. *Plant cell reports* 21**,** 814-820.

Jensen, G., Hansen, B., Eilenberg, J., and Mahillon, J. (2003). The hidden lifestyles of *Bacillus cereus* and relatives. *Environmental microbiology* 5**,** 631-640.

Khan, M., and Patel, C. (2007). Plant growth promoting effect of *Bacillus firmus* strain NARS1 isolated from central Himalayan region of India on *Cicer arientnum* at low temperature. *Egypt Afr Crop Sci Soc* 8**,** 1179-1181.

Lidstrom, M.E., and Chistoserdova, L. (2002). Plants in the pink: cytokinin production by *Methylobacterium*. *Journal of bacteriology* 184**,** 1818-1818.

Lin, L., Guo, W., Xing, Y., Zhang, X., Li, Z., Hu, C., Li, S., Li, Y., and An, Q. (2012). The actinobacterium *Microbacterium* sp. 16SH accepts pBBR1-based pPROBE vectors, forms biofilms, invades roots, and fixes N2 associated with micropropagated sugarcane plants. *Applied microbiology and biotechnology* 93**,** 1185-1195.

Mandic-Mulec, I., and Prosser, J.I. (2011). "Diversity of endospore-forming bacteria in soil: characterization and driving mechanisms," in *Endospore-forming Soil Bacteria*. Springer), 43.

Mcspadden Gardener, B.B. (2004). Ecology of *Bacillus* and *Paenibacillus* spp. in agricultural systems. *Phytopathology* 94**,** 1252-1258.

Munoz-Price, L.S., and Weinstein, R.A. (2008). *Acinetobacter* infection. *New England Journal of Medicine* 358**,** 1271-1281.

Odutayo, O., Amusa, N., Okutade, O., and Ogunsanwo, Y. (2007). Sources of microbial contamination in tissue culture laboratories in southwestern Nigeria. *Afr J Agric Res* 2**,** 67-72.

Rokhbakhsh-Zamin, F., Sachdev, D., Kazemi-Pour, N., Engineer, A., Pardesi, K.R., Zinjarde, S., Dhakephalkar, P.K., and Chopade, B.A. (2011). Characterization of plant-growth-promoting traits of *Acinetobacter* species isolated from rhizosphere of *Pennisetum glaucum*. *Journal of microbiology and biotechnology* 21**,** 556-566.

Schmidt, S. (2010). *Functional investigation of methanol dehydrogenase-like protein XoxF in Methylobacterium extorquens AM1 [Dissertation]: ETH Zurich.* Doctoral, ETH Zürich.

Silo-Suh, L.A., Lethbridge, B.J., Raffel, S.J., He, H., Clardy, J., and Handelsman, J. (1994). Biological activities of two fungistatic antibiotics produced by *Bacillus cereus* UW85. *Applied and environmental microbiology* 60**,** 2023-2030.

Thomas, P., Kumari, S., Swarna, G.K., and Gowda, T. (2007). Papaya shoot tip associated endophytic bacteria isolated from *in vitro* cultures and host-endophyte interaction *in vitro* and *in vivo*. *Canadian journal of microbiology* 53**,** 380-390.

Thomas, P., Panicker, B., Janakiram, T., and Sathyanarayana, B. (2009). *In vitro* propagation of'Arka Ravi'chrysanthemum on growth regulator-free medium harbouring endophytic bacteria. *Journal of Horticultural Science and Biotechnology* 84**,** 653-659.

Thomas, P., and Soly, T.A. (2009). Endophytic bacteria associated with growing shoot tips of banana (*Musa* sp.) cv. Grand Naine and the affinity of endophytes to the host. *Microbial ecology* 58**,** 952-964.

Thomas, P., Swarna, G.K., Roy, P.K., and Patil, P. (2008). Identification of culturable and originally non-culturable endophytic bacteria isolated from shoot tip cultures of banana cv. Grand Naine. *Plant Cell, Tissue and Organ Culture* 93**,** 55-63.

Usha, R. (2011). *Antibiotic production, Purification, Characterization and Biological activity OF newly isolated Streptomyces parvulus KUAP106.* Microbiology, Ph. D. University of Karpagam.

Usha, R., Ananthaselvi, P., Venil, C., and Palaniswamy, M. (2010). Antimicrobial and antiangiogenesis activity of *Streptomyces parvulus* KUAP106 from mangrove soil. *Eur J Biol Sci* 2**,** 77-83.

Winn, W.C., Allen, S., Janda, W., Koneman, E.W., Procop, G., Schreckenberger, P., and Woods, G. (2006). "Aerobic and facultative gram-positive bacilli," in *Koneman's color atlas and textbook of diagnostic microbiology,* ed. E.W. Koneman. 6 ed: Lippincott Williams & Wilkins), 823-825.

Zahar Haichar, F., Marol, C., Berge, O., Rangel-Castro, J.I., Prosser, J.I., Balesdent, J., Heulin, T., and Achouak, W. (2008). Plant host habitat and root exudates shape soil bacterial community structure. *The ISME journal* 2**,** 1221-1230.

Zawadzka, M., Orlikowska, T., Sobiczewski, P., Mikicinski, A., Sulikowska, M., and Zenkteler, E. (2009). The control of bacterial contaminations during in vitro shoot multiplication. *Acta Biologica Cracoviensia. Series Botanica. Supplement* 51.

Zhang, X., Clark, C.A., and Pettis, G.S. (2003). Interstrain inhibition in the sweet potato pathogen *Streptomyces ipomoeae*: purification and characterization of a highly specific bacteriocin and cloning of its structural gene. *Applied and environmental microbiology* 69**,** 2201-2208
